# Crystal structure of Sc_1.91_In_1.39_Mo_15_Se_19_, containing Mo_6_ and Mo_9_ clusters

**DOI:** 10.1107/S2056989015010634

**Published:** 2015-06-10

**Authors:** Patrick Gougeon, Philippe Gall, Diala Salloum

**Affiliations:** a Sciences Chimiques de Rennes, UMR CNRS No. 6226, Université de Rennes I - INSA Rennes, Avenue du Général Leclerc, 35042 Rennes CEDEX, France

**Keywords:** crystal structure, Mo clusters, reduced molybdenum selenide, monovalent indium

## Abstract

The structure of scandium indium penta­deca­molybdenum nona­deca­selenide, Sc_1.91_In_1.39_Mo_15_Se_19_, is isotypic with In_2.9_Mo_15_Se_19_ [Grüttner *et al.* (1979 ▸). *Acta Cryst.* B**35**, 285–292]. It is characterized by two cluster units Mo_6_Se^*i*^_8_Se^*a*^_6_ and Mo_9_Se^*i*^_11_Se^*a*^_6_ (where *i* represents inner and *a* apical atoms) that are present in a 1:1 ratio. The cluster units are centered at Wyckoff positions 2*b* and 2*c* and have point-group symmetry -3 and -6, respectively. The clusters are inter­connected through additional Mo—Se bonds. Sc—Se and In—Se bonds complete the structural set-up. In the title compound, the Sc^3+^ cations replace the trivalent indium atoms present in In_2.9_Mo_15_Se_19_, and a deficiency is observed at the monovalent indium site. One Mo, one Se and the Sc atom are situated on mirror planes, whereas two other Se atoms and the In atom are situated on threefold rotation axes.

## Abstract 2

The crystal structure of the title compound consists of an equal mixture of the cluster units Mo_6_Se^*i*^_8_Se^*a*^_6_ and Mo_9_Se^*i*^_11_Se^*a*^_6_ separated by deficient In^+^ and Sc^3+^ cations.

## Chemical context   

From a crystal–chemical point of view, reduced molybdenum selenides In_3_Mo_15_Se_19_ (Grüttner *et al.*, 1979 ▸) constitute an inter­esting family of compounds. Indeed, their crystal structures contain an equal mixture of Mo_6_ and Mo_9_ cluster units with the In atoms occupying two crystallographically different positions depending on their formal oxidation state of +1 or +3. Inter­est in these Mo cluster compounds also lies in their physical properties because they become superconductors with high critical magnetic fields at about 4 K (Seeber *et al.*, 1979 ▸). Recently, we have shown that the In^3+^ cation can be replaced by other trivalent cations such as Ho^3+^ (resulting in a compound with composition Ho_0.76_In_1.68_Mo_15_Se_19_; Salloum *et al.*, 2006 ▸) or V^3+^ (V_1.42_In_1.83_Mo_15_Se_19_; Gougeon *et al.*, 2010 ▸), and the In^+^ cation by K^+^ (In_0.87_K_2_Mo_15_Se_19_; Salloum *et al.*, 2007 ▸). We present here the crystal structure of Sc_1.91_In_1.39_Mo_15_Se_19_ in which scandium atoms replace the trivalent indium atoms.

## Structural commentary   

**Figure 1:**
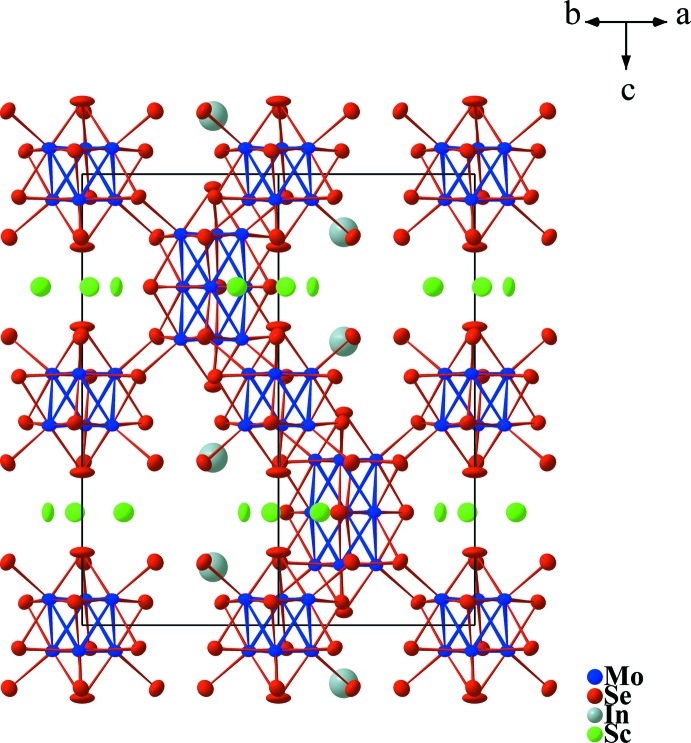
View of the crystal structure of Sc_1.91_In_1.39_Mo_15_Se_19_ along [110]. Displacement ellipsoids are drawn at the 97% probability level.

The Mo–Se framework of the title compound consists of the cluster units Mo_6_Se^*i*^_8_Se^*a*^_6_ and Mo_9_Se^*i*^_11_Se^*a*^_6_ in an 1:1 ratio (for details of the *i*- and *a*-type ligand notation, see: Schäfer & von Schnering, 1964 ▸). Both cluster units are inter­connected through additional Mo—Se bonds (Table 1 ▸, Figs. 1 ▸ and 2 ▸). The first unit can be described as an Mo_6_ octa­hedron surrounded by eight face-capping inner Se^*i*^ and six apical Se^*a*^ ligands. The Mo_9_ cluster is surrounded by 11 Se^*i*^ atoms capping one or two faces of the bi­octa­hedron and six Se^*a*^ ligands above the apical Mo atoms. The Mo_6_Se^*i*^_8_Se^*a*^_6_ and Mo_9_Se^*i*^_11_Se^*a*^_6_ units are centered at Wyckoff positions 2*b* and 2*c* and have point-group symmetry 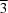 and 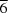, respectively. The Mo—Mo distances within the Mo_6_ cluster are 2.6995 (6) Å for the distances of the Mo triangles formed by the Mo1 atoms related through the threefold axis, and 2.7179 (5) Å for the distances between these triangles. The Mo—Mo distances within the Mo_9_ clusters are 2.6460 (6) and 2.7127 (8) Å in the triangles formed by the atoms Mo2 and Mo3, respectively, and 2.7196 (4) and 2.7675 (4) Å for those between the Mo2_3_ and Mo3_3_ triangles. The Se atoms bridge either one (Se1, Se2, Se4 and Se5) or two (Se3) triangular faces of the Mo clusters. Moreover, atoms Se1 and Se2 are linked to an Mo atom of a neighboring cluster. The Mo—Se bond lengths range from 2.5480 (6) to 2.6531 (5) Å within the Mo_6_Se^*i*^_8_Se^*a*^_6_ unit, and from 2.5290 (6) to 2.6966 (4) Å within the Mo_9_Se^*i*^_11_Se^*a*^_6_ unit. Each Mo_9_Se^*i*^_11_Se^*a*^_6_ cluster is inter­connected by six Mo_6_Se^*i*^_8_Se^*a*^_6_ units (and *vice versa*) *via* Mo2—Se1 bonds (and Mo1—Se2 bonds, respectively), forming the three-dimensional Mo–Se framework, the connectivity formula of which is Mo_9_Se^*i*^_5_Se^*i−a*^_6/2_Se^*a−i*^_6/2_, Mo_6_Se^*i*^_2_Se^*i−a*^_6/2_Se^*a−i*^_6/2_. It results from this arrangement that the shortest inter­cluster Mo1—Mo2 distance is 3.4361 (5) Å, indicating only weak metal⋯metal inter­actions.

Comparison of the Mo—Mo and Mo—Se distances with those of the other substituted compounds Ho_0.76_In_1.68_Mo_15_Se_19_, In_0.87_K_2_Mo_15_Se_19_ and V_1.42_In_1.83_Mo_15_Se_19_ does not reveal great differences although the cationic charges are different in the four compounds. The In^+^ cations are surrounded by seven Se atoms, forming a distorted tricapped tetra­hedron as in In_2.9_Mo_15_Se_19_. The Se5 and Se2 atoms forming the tetra­hedron are at 3.0662 (13) and 3.1273 (4) Å from the In^+^ cation, and the capping Se1 atoms are at 3.4912 (6) Å. While in In_2.9_Mo_15_Se_19_ the monovalent In site is fully occupied, in the title compound it is only has 69.5 (3)% occupancy. This deficiency probably results from the higher temperature used during the crystal-growth process, which led to a loss of indium and selenium because of the high volatility of these elements at 1773 K. The Sc^3+^ cations, as the In^3+^ cations in the In_3_Mo_15_Se_19_ compounds, occupy partially at 63.8 (6)% a triangular group of distorted octa­hedral cavities, which are formed by two Mo_6_Se^*i*^_8_Se^*a*^_6_ and three Mo_9_Se^*i*^_11_Se^*a*^_6_ units, around the threefold rotation axis. The Sc—Se distances are in the 2.5691 (15)–2.931 (2) Å range.

## Synthesis and crystallization   

Single crystals of Sc_1.91_In_1.39_Mo_15_Se_19_ were obtained from a mixture of Sc_2_Se_3_, MoSe_2_, InSe and Mo with a nominal composition Sc_2_In_2_Mo_15_Se_19_. Before use, Mo powder was reduced under H_2_ flowing gas at 1273 K for ten h in order to eliminate any trace of oxygen. The binaries Sc_2_Se_3_, MoSe_2_, InSe were obtained by heating stoichiometric mixtures of the elements in sealed evacuated silica tubes for about two days. All handling of materials was performed in an argon-filled glove box. The initial mixture (*ca* 5 g) was cold pressed and loaded into a molybdenum crucible, which was sealed under a low argon pressure using an arc welding system. The charge was heated at the rate of 300 K h^−1^ up to 1773 K, the temperature which was held for 48 h, then cooled at 100 K h^−1^ down to 1373 K and finally furnace cooled.

## Refinement   

Crystal data, data collection and structure refinement details are summarized in Table 2 ▸. The highest and lowest remaining electron densities are located 0.66 and 0.62 Å from the In site, respectively. Refinement of the occupancy factors of the Sc and In atoms led to the final composition Sc_1.914 (12)_In_1.390 (6)_Mo_15_Se_19_.

## Supplementary Material

Crystal structure: contains datablock(s) I, global. DOI: 10.1107/S2056989015010634/wm5164sup1.cif

Structure factors: contains datablock(s) I. DOI: 10.1107/S2056989015010634/wm5164Isup2.hkl

CCDC reference: 1404496

Additional supporting information:  crystallographic information; 3D view; checkCIF report

## Figures and Tables

**Figure 2:**
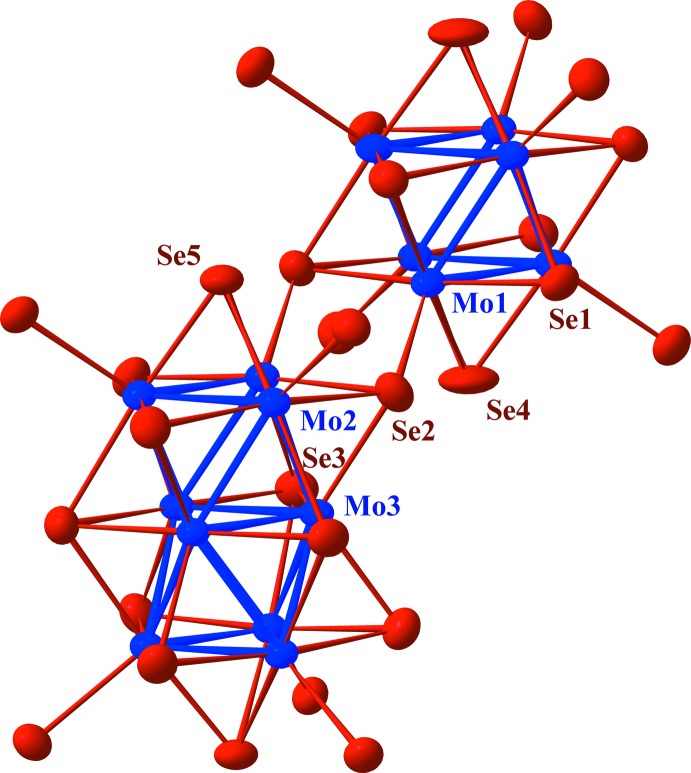
Plot showing the atom-numbering scheme and the inter­unit linkage of the Mo_9_Se_11_Se_6_ and Mo_6_Se_8_Se_6_ cluster units. Displacement ellipsoids are drawn at the 97% probability level.

**Table 1:** Selected bond lengths ()

Mo1Se4	2.5480(6)	Mo3Se2	2.5780(5)
Mo1Se1^i^	2.5488(5)	Mo3Se3^iii^	2.5884(7)
Mo1Se1	2.5749(5)	Mo3Se3	2.5900(7)
Mo1Se1^ii^	2.6145(5)	Mo3Mo3^iii^	2.7127(8)
Mo1Se2	2.6531(5)	InSe5	3.0662(13)
Mo1Mo1^ii^	2.6995(6)	InSe2^vii^	3.1273(4)
Mo1Mo1^i^	2.7179(5)	InSe2^viii^	3.1273(4)
Mo2Se5	2.5290(6)	InSe2^i^	3.1273(4)
Mo2Se2	2.5931(5)	InSe1^vii^	3.4912(6)
Mo2Se2^iii^	2.6275(5)	InSe1^i^	3.4912(6)
Mo2Mo2^iv^	2.6460(6)	InSe1^viii^	3.4912(6)
Mo2Se1^v^	2.6581(5)	ScSe4^vi^	2.5691(15)
Mo2Se3^iii^	2.6965(4)	ScSe3^ii^	2.696(2)
Mo2Mo3^iii^	2.7196(4)	ScSe2^ix^	2.8056(12)
Mo2Mo3	2.7675(4)	ScSe2^x^	2.8056(12)
Mo3Se2^vi^	2.5780(5)	ScSe3^ix^	2.931(2)

Symmetry codes: (i) 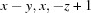; (ii) 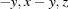; (iii) 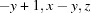; (iv) 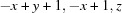; (v) 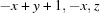; (vi) 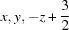; (vii) 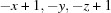; (viii) 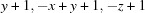; (ix) 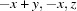; (x) 
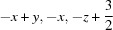
.

**Table 2:** Experimental details

Crystal data	
Chemical formula	Sc_1.91_In_1.39_Mo_15_Se_19_
*M*_r_	3185.04
Crystal system, space group	Hexagonal, *P*6_3_/*m*
Temperature (K)	293
*a*, *c* ()	9.7530(2), 19.3977(2)
*V* (^3^)	1597.93(7)
*Z*	2
Radiation type	Mo *K*
(mm^1^)	28.65
Crystal size (mm)	0.06 0.05 0.04

Data collection	
Diffractometer	Nonius KappaCCD
Absorption correction	Analytical (de Meulenaar Tompa, 1965 ▸)
*T*_min_, *T*_max_	0.279, 0.424
No. of measured, independent and observed [*I* > 2(*I*)] reflections	31413, 2414, 1847
*R*_int_	0.073
(sin /)_max_ (^1^)	0.807

Refinement	
*R*[*F* ^2^ > 2(*F* ^2^)], *wR*(*F* ^2^), *S*	0.031, 0.063, 1.08
No. of reflections	2414
No. of parameters	67
_max_, _min_ (e ^3^)	2.22, 1.97

Computer programs: *COLLECT* (Nonius, 1998 ▸), *EVALCCD* (Duisenberg *et al.*, 2003 ▸), *SIR97* (Altomare *et al.*, 1999 ▸), *SHELXL2014*/6 (Sheldrick, 2015 ▸) and *DIAMOND* (Bergerhoff, 1996 ▸).

## supplementary crystallographic information

### Crystal data

**Table:**

Sc_1.91_In_1.39_Mo_15_Se_19_	*F*(000) = 2769
*M**_r_* = 3185.04	*D*_x_ = 6.620 Mg m^−^^3^
Hexagonal, *P*6_3_/*m*	Mo *K*α radiation, λ = 0.71069 Å
*a* = 9.7530 (2) Å	µ = 28.65 mm^−^^1^
*c* = 19.3977 (2) Å	*T* = 293 K
*V* = 1597.93 (7) Å^3^	Irregular block, black
*Z* = 2	0.06 × 0.05 × 0.04 mm

### Data collection

**Table:**

Nonius KappaCCD diffractometer	2414 independent reflections
Radiation source: fine-focus sealed tube	1847 reflections with *I* > 2σ(*I*)
Graphite monochromator	*R*_int_ = 0.073
φ scans (κ = 0) + additional ω scans	θ_max_ = 35.0°, θ_min_ = 2.4°
Absorption correction: analytical (de Meulenaar & Tompa, 1965)	*h* = −13→15
*T*_min_ = 0.279, *T*_max_ = 0.424	*k* = −15→13
31413 measured reflections	*l* = −31→26

### Refinement

**Table:**

Refinement on *F*^2^	0 restraints
Least-squares matrix: full	*w* = 1/[σ^2^(*F*_o_^2^) + (0.0228*P*)^2^ + 7.5263*P*] where *P* = (*F*_o_^2^ + 2*F*_c_^2^)/3
*R*[*F*^2^ > 2σ(*F*^2^)] = 0.031	(Δ/σ)_max_ = 0.002
*wR*(*F*^2^) = 0.063	Δρ_max_ = 2.22 e Å^−^^3^
*S* = 1.08	Δρ_min_ = −1.96 e Å^−^^3^
2414 reflections	Extinction correction: *SHELXL*, Fc^*^=kFc[1+0.001xFc^2^λ^3^/sin(2θ)]^-1/4^
67 parameters	Extinction coefficient: 0.00065 (4)

### Special details

**Table:**

Geometry. All esds (except the esd in the dihedral angle between two l.s. planes) are estimated using the full covariance matrix. The cell esds are taken into account individually in the estimation of esds in distances, angles and torsion angles; correlations between esds in cell parameters are only used when they are defined by crystal symmetry. An approximate (isotropic) treatment of cell esds is used for estimating esds involving l.s. planes.

### Fractional atomic coordinates and isotropic or equivalent isotropic displacement parameters (Å^2^)

**Table:**

	*x*	*y*	*z*	*U*_iso_*/*U*_eq_	Occ. (<1)
Mo1	0.16732 (4)	0.01628 (4)	0.55740 (2)	0.00813 (7)	
Mo2	0.68442 (4)	0.18633 (4)	0.63321 (2)	0.00769 (7)	
Mo3	0.51261 (5)	0.16694 (5)	0.7500	0.00748 (8)	
Se1	0.03573 (5)	−0.28735 (5)	0.55124 (2)	0.01007 (8)	
Se2	0.37937 (5)	0.00705 (5)	0.64001 (2)	0.01114 (9)	
Se3	0.34923 (7)	0.30997 (7)	0.7500	0.01107 (11)	
Se4	0.0000	0.0000	0.66131 (3)	0.01933 (16)	
Se5	0.6667	0.3333	0.52931 (3)	0.01116 (13)	
In	0.6667	0.3333	0.37124 (6)	0.0358 (4)	0.695 (3)
Sc	−0.2115 (2)	−0.1745 (2)	0.7500	0.0143 (5)	0.638 (6)

### Atomic displacement parameters (Å^2^)

**Table:**

	*U*^11^	*U*^22^	*U*^33^	*U*^12^	*U*^13^	*U*^23^
Mo1	0.01005 (15)	0.00828 (15)	0.00598 (13)	0.00453 (12)	0.00026 (9)	0.00006 (9)
Mo2	0.00846 (14)	0.00829 (14)	0.00626 (12)	0.00415 (12)	−0.00015 (9)	−0.00030 (9)
Mo3	0.0087 (2)	0.0085 (2)	0.00563 (16)	0.00458 (16)	0.000	0.000
Se1	0.01139 (18)	0.00980 (18)	0.00960 (15)	0.00572 (15)	0.00112 (12)	0.00197 (12)
Se2	0.00992 (18)	0.01101 (19)	0.01187 (16)	0.00477 (14)	−0.00272 (12)	−0.00153 (12)
Se3	0.0098 (3)	0.0142 (3)	0.0104 (2)	0.0069 (2)	0.000	0.000
Se4	0.0264 (3)	0.0264 (3)	0.0053 (3)	0.01318 (13)	0.000	0.000
Se5	0.0136 (2)	0.0136 (2)	0.0063 (2)	0.00679 (10)	0.000	0.000
In	0.0355 (5)	0.0355 (5)	0.0363 (6)	0.0178 (2)	0.000	0.000
Sc	0.0176 (10)	0.0064 (8)	0.0159 (8)	0.0038 (7)	0.000	0.000

### Geometric parameters (Å, º)

**Table:**

Mo1—Se4	2.5480 (6)	Se3—Sc^ii^	2.931 (2)
Mo1—Se1^i^	2.5488 (5)	Se4—Mo1^iii^	2.5479 (6)
Mo1—Se1	2.5749 (5)	Se4—Mo1^ii^	2.5479 (6)
Mo1—Se1^ii^	2.6145 (5)	Se4—Sc^iii^	2.5691 (15)
Mo1—Se2	2.6531 (5)	Se4—Sc^ii^	2.5691 (15)
Mo1—Mo1^ii^	2.6995 (6)	Se4—Sc	2.5691 (15)
Mo1—Mo1^iii^	2.6995 (6)	Se5—Mo2^v^	2.5290 (6)
Mo1—Mo1^i^	2.7179 (5)	Se5—Mo2^vi^	2.5290 (6)
Mo1—Mo1^iv^	2.7179 (5)	In—Se5	3.0662 (13)
Mo2—Se5	2.5290 (6)	In—Se2^xi^	3.1273 (4)
Mo2—Se2	2.5931 (5)	In—Se2^xii^	3.1273 (4)
Mo2—Se2^v^	2.6275 (5)	In—Se2^i^	3.1273 (4)
Mo2—Mo2^vi^	2.6460 (6)	In—Se1^xi^	3.4912 (6)
Mo2—Mo2^v^	2.6460 (6)	In—Se1^i^	3.4912 (6)
Mo2—Se1^vii^	2.6581 (5)	In—Se1^xii^	3.4912 (6)
Mo2—Se3^v^	2.6965 (4)	In—Se3^iv^	4.2657 (8)
Mo2—Mo3^v^	2.7196 (4)	In—Se3^xiii^	4.2657 (8)
Mo2—Mo3	2.7675 (4)	In—Se3^xiv^	4.2657 (8)
Mo3—Se2^viii^	2.5780 (5)	In—Sc^xv^	4.5565 (18)
Mo3—Se2	2.5780 (5)	In—Sc^xvi^	4.5565 (18)
Mo3—Se3^v^	2.5884 (7)	In—Sc^xvii^	4.5565 (18)
Mo3—Se3	2.5900 (7)	Sc—Se4^viii^	2.5691 (15)
Mo3—Mo3^v^	2.7127 (8)	Sc—Se3^ii^	2.696 (2)
Mo3—Mo3^vi^	2.7127 (8)	Sc—Se2^iii^	2.8056 (12)
Mo3—Mo2^ix^	2.7196 (4)	Sc—Se2^xviii^	2.8056 (12)
Mo3—Mo2^vi^	2.7196 (4)	Sc—Mo3^iii^	2.8760 (18)
Mo3—Mo2^viii^	2.7675 (4)	Sc—Se3^iii^	2.931 (2)
Mo3—Sc^ii^	2.8760 (18)	Sc—Sc^iii^	3.305 (3)
Se1—Mo1^iv^	2.5488 (5)	Sc—Sc^ii^	3.305 (3)
Se1—Mo1^iii^	2.6145 (5)	Sc—Mo1^xviii^	3.7775 (4)
Se1—Mo2^x^	2.6581 (5)	Sc—Mo1^iii^	3.7775 (4)
Se2—Mo2^vi^	2.6275 (5)	Sc—Mo2^x^	4.0216 (15)
Se2—Sc^ii^	2.8056 (12)	Sc—Mo2^xix^	4.0216 (15)
Se3—Mo3^vi^	2.5884 (7)	Sc—Se1^xviii^	4.3993 (10)
Se3—Mo2^ix^	2.6966 (4)	Sc—Se1^iii^	4.3993 (10)
Se3—Mo2^vi^	2.6966 (4)	Sc—In^xvi^	4.5564 (18)
Se3—Sc^iii^	2.696 (2)		
			
Se4—Mo1—Se1^i^	176.406 (19)	Mo2^ix^—Mo3—Mo2	145.97 (2)
Se4—Mo1—Se1	91.698 (13)	Mo2^vi^—Mo3—Mo2	57.653 (13)
Se1^i^—Mo1—Se1	89.030 (13)	Se2^viii^—Mo3—Mo2^viii^	57.907 (12)
Se4—Mo1—Se1^ii^	90.788 (13)	Se2—Mo3—Mo2^viii^	145.46 (2)
Se1^i^—Mo1—Se1^ii^	88.161 (13)	Se3^v^—Mo3—Mo2^viii^	60.349 (12)
Se1—Mo1—Se1^ii^	174.05 (2)	Se3—Mo3—Mo2^viii^	118.538 (13)
Se4—Mo1—Se2	90.338 (16)	Mo3^v^—Mo3—Mo2^viii^	59.495 (13)
Se1^i^—Mo1—Se2	93.220 (16)	Mo3^vi^—Mo3—Mo2^viii^	88.794 (12)
Se1—Mo1—Se2	86.430 (16)	Mo2^ix^—Mo3—Mo2^viii^	57.654 (13)
Se1^ii^—Mo1—Se2	98.964 (16)	Mo2^vi^—Mo3—Mo2^viii^	145.97 (2)
Se4—Mo1—Mo1^ii^	58.012 (9)	Mo2—Mo3—Mo2^viii^	109.89 (2)
Se1^i^—Mo1—Mo1^ii^	118.654 (14)	Se2^viii^—Mo3—Sc^ii^	61.629 (19)
Se1—Mo1—Mo1^ii^	119.304 (17)	Se2—Mo3—Sc^ii^	61.629 (19)
Se1^ii^—Mo1—Mo1^ii^	57.940 (16)	Se3^v^—Mo3—Sc^ii^	118.59 (5)
Se2—Mo1—Mo1^ii^	137.271 (14)	Se3—Mo3—Sc^ii^	64.60 (4)
Se4—Mo1—Mo1^iii^	58.012 (9)	Mo3^v^—Mo3—Sc^ii^	177.02 (5)
Se1^i^—Mo1—Mo1^iii^	119.634 (13)	Mo3^vi^—Mo3—Sc^ii^	122.98 (5)
Se1—Mo1—Mo1^iii^	59.376 (17)	Mo2^ix^—Mo3—Sc^ii^	91.85 (3)
Se1^ii^—Mo1—Mo1^iii^	117.870 (16)	Mo2^vi^—Mo3—Sc^ii^	91.85 (3)
Se2—Mo1—Mo1^iii^	129.426 (16)	Mo2—Mo3—Sc^ii^	119.536 (18)
Mo1^ii^—Mo1—Mo1^iii^	60.0	Mo2^viii^—Mo3—Sc^ii^	119.536 (18)
Se4—Mo1—Mo1^i^	118.212 (12)	Mo1^iv^—Se1—Mo1	64.071 (16)
Se1^i^—Mo1—Mo1^i^	58.430 (15)	Mo1^iv^—Se1—Mo1^iii^	63.509 (16)
Se1—Mo1—Mo1^i^	117.048 (15)	Mo1—Se1—Mo1^iii^	62.685 (17)
Se1^ii^—Mo1—Mo1^i^	57.068 (11)	Mo1^iv^—Se1—Mo2^x^	131.401 (19)
Se2—Mo1—Mo1^i^	140.439 (16)	Mo1—Se1—Mo2^x^	128.246 (18)
Mo1^ii^—Mo1—Mo1^i^	60.224 (8)	Mo1^iii^—Se1—Mo2^x^	81.335 (15)
Mo1^iii^—Mo1—Mo1^i^	90.0	Mo3—Se2—Mo2	64.712 (15)
Se4—Mo1—Mo1^iv^	118.212 (12)	Mo3—Se2—Mo2^vi^	62.984 (14)
Se1^i^—Mo1—Mo1^iv^	59.423 (15)	Mo2—Se2—Mo2^vi^	60.903 (17)
Se1—Mo1—Mo1^iv^	57.499 (11)	Mo3—Se2—Mo1	130.17 (2)
Se1^ii^—Mo1—Mo1^iv^	116.606 (15)	Mo2—Se2—Mo1	126.687 (18)
Se2—Mo1—Mo1^iv^	132.232 (15)	Mo2^vi^—Se2—Mo1	81.187 (15)
Mo1^ii^—Mo1—Mo1^iv^	90.0	Mo3—Se2—Sc^ii^	64.42 (3)
Mo1^iii^—Mo1—Mo1^iv^	60.224 (8)	Mo2—Se2—Sc^ii^	129.13 (3)
Mo1^i^—Mo1—Mo1^iv^	59.550 (15)	Mo2^vi^—Se2—Sc^ii^	95.44 (4)
Se5—Mo2—Se2	92.412 (13)	Mo1—Se2—Sc^ii^	87.53 (3)
Se5—Mo2—Se2^v^	91.604 (13)	Mo3^vi^—Se3—Mo3	63.18 (2)
Se2—Mo2—Se2^v^	174.13 (2)	Mo3^vi^—Se3—Mo2^ix^	63.118 (13)
Se5—Mo2—Mo2^vi^	58.457 (9)	Mo3—Se3—Mo2^ix^	61.881 (13)
Se2—Mo2—Mo2^vi^	60.191 (17)	Mo3^vi^—Se3—Mo2^vi^	63.118 (13)
Se2^v^—Mo2—Mo2^vi^	118.823 (16)	Mo3—Se3—Mo2^vi^	61.881 (14)
Se5—Mo2—Mo2^v^	58.457 (9)	Mo2^ix^—Se3—Mo2^vi^	114.31 (2)
Se2—Mo2—Mo2^v^	120.106 (17)	Mo3^vi^—Se3—Sc^iii^	162.59 (5)
Se2^v^—Mo2—Mo2^v^	58.906 (16)	Mo3—Se3—Sc^iii^	134.23 (5)
Mo2^vi^—Mo2—Mo2^v^	60.0	Mo2^ix^—Se3—Sc^iii^	121.404 (15)
Se5—Mo2—Se1^vii^	90.196 (15)	Mo2^vi^—Se3—Sc^iii^	121.404 (15)
Se2—Mo2—Se1^vii^	85.764 (16)	Mo3^vi^—Se3—Sc^ii^	125.62 (4)
Se2^v^—Mo2—Se1^vii^	98.510 (16)	Mo3—Se3—Sc^ii^	62.43 (4)
Mo2^vi^—Mo2—Se1^vii^	129.579 (16)	Mo2^ix^—Se3—Sc^ii^	91.14 (2)
Mo2^v^—Mo2—Se1^vii^	137.688 (14)	Mo2^vi^—Se3—Sc^ii^	91.14 (2)
Se5—Mo2—Se3^v^	175.172 (19)	Sc^iii^—Se3—Sc^ii^	71.79 (8)
Se2—Mo2—Se3^v^	85.424 (18)	Mo1^iii^—Se4—Mo1^ii^	63.974 (19)
Se2^v^—Mo2—Se3^v^	90.244 (18)	Mo1^iii^—Se4—Mo1	63.975 (19)
Mo2^vi^—Mo2—Se3^v^	116.797 (15)	Mo1^ii^—Se4—Mo1	63.974 (19)
Mo2^v^—Mo2—Se3^v^	119.114 (14)	Mo1^iii^—Se4—Sc^iii^	148.00 (4)
Se1^vii^—Mo2—Se3^v^	93.942 (17)	Mo1^ii^—Se4—Sc^iii^	95.16 (3)
Se5—Mo2—Mo3^v^	120.527 (17)	Mo1—Se4—Sc^iii^	130.64 (4)
Se2—Mo2—Mo3^v^	116.554 (16)	Mo1^iii^—Se4—Sc^ii^	130.64 (4)
Se2^v^—Mo2—Mo3^v^	57.619 (13)	Mo1^ii^—Se4—Sc^ii^	148.00 (4)
Mo2^vi^—Mo2—Mo3^v^	91.213 (12)	Mo1—Se4—Sc^ii^	95.16 (3)
Mo2^v^—Mo2—Mo3^v^	62.083 (12)	Sc^iii^—Se4—Sc^ii^	80.06 (5)
Se1^vii^—Mo2—Mo3^v^	138.895 (18)	Mo1^iii^—Se4—Sc	95.16 (3)
Se3^v^—Mo2—Mo3^v^	57.133 (16)	Mo1^ii^—Se4—Sc	130.64 (4)
Se5—Mo2—Mo3	118.709 (16)	Mo1—Se4—Sc	148.00 (4)
Se2—Mo2—Mo3	57.381 (13)	Sc^iii^—Se4—Sc	80.06 (5)
Se2^v^—Mo2—Mo3	116.832 (16)	Sc^ii^—Se4—Sc	80.06 (5)
Mo2^vi^—Mo2—Mo3	60.264 (12)	Mo2^v^—Se5—Mo2	63.086 (18)
Mo2^v^—Mo2—Mo3	90.162 (12)	Mo2^v^—Se5—Mo2^vi^	63.086 (18)
Se1^vii^—Mo2—Mo3	131.676 (18)	Mo2—Se5—Mo2^vi^	63.086 (18)
Se3^v^—Mo2—Mo3	56.533 (15)	Se4^viii^—Sc—Se4	84.08 (6)
Mo3^v^—Mo2—Mo3	59.250 (17)	Se4^viii^—Sc—Se3^ii^	88.06 (5)
Se2^viii^—Mo3—Se2	111.71 (3)	Se4—Sc—Se3^ii^	88.06 (5)
Se2^viii^—Mo3—Se3^v^	87.997 (16)	Se4^viii^—Sc—Se2^iii^	163.50 (8)
Se2—Mo3—Se3^v^	87.997 (16)	Se4—Sc—Se2^iii^	86.578 (17)
Se2^viii^—Mo3—Se3	93.784 (17)	Se3^ii^—Sc—Se2^iii^	105.20 (5)
Se2—Mo3—Se3	93.784 (17)	Se4^viii^—Sc—Se2^xviii^	86.579 (17)
Se3^v^—Mo3—Se3	176.82 (2)	Se4—Sc—Se2^xviii^	163.51 (8)
Se2^viii^—Mo3—Mo3^v^	117.324 (17)	Se3^ii^—Sc—Se2^xviii^	105.20 (5)
Se2—Mo3—Mo3^v^	117.324 (17)	Se2^iii^—Sc—Se2^xviii^	99.02 (6)
Se3^v^—Mo3—Mo3^v^	58.44 (2)	Se4^viii^—Sc—Mo3^iii^	120.92 (5)
Se3—Mo3—Mo3^v^	118.38 (2)	Se4—Sc—Mo3^iii^	120.92 (5)
Se2^viii^—Mo3—Mo3^vi^	120.613 (15)	Se3^ii^—Sc—Mo3^iii^	138.83 (8)
Se2—Mo3—Mo3^vi^	120.614 (15)	Se2^iii^—Sc—Mo3^iii^	53.95 (3)
Se3^v^—Mo3—Mo3^vi^	118.44 (2)	Se2^xviii^—Sc—Mo3^iii^	53.95 (3)
Se3—Mo3—Mo3^vi^	58.38 (2)	Se4^viii^—Sc—Se3^iii^	83.19 (5)
Mo3^v^—Mo3—Mo3^vi^	60.0	Se4—Sc—Se3^iii^	83.19 (5)
Se2^viii^—Mo3—Mo2^ix^	59.397 (12)	Se3^ii^—Sc—Se3^iii^	168.21 (8)
Se2—Mo3—Mo2^ix^	150.43 (2)	Se2^iii^—Sc—Se3^iii^	82.23 (4)
Se3^v^—Mo3—Mo2^ix^	118.002 (13)	Se2^xviii^—Sc—Se3^iii^	82.23 (4)
Se3—Mo3—Mo2^ix^	60.986 (12)	Mo3^iii^—Sc—Se3^iii^	52.97 (3)
Mo3^v^—Mo3—Mo2^ix^	89.795 (12)	Se4^viii^—Sc—Sc^iii^	49.97 (2)
Mo3^vi^—Mo3—Mo2^ix^	61.256 (13)	Se4—Sc—Sc^iii^	49.97 (2)
Se2^viii^—Mo3—Mo2^vi^	150.43 (2)	Se3^ii^—Sc—Sc^iii^	57.39 (6)
Se2—Mo3—Mo2^vi^	59.397 (12)	Se2^iii^—Sc—Sc^iii^	129.92 (3)
Se3^v^—Mo3—Mo2^vi^	118.002 (13)	Se2^xviii^—Sc—Sc^iii^	129.92 (3)
Se3—Mo3—Mo2^vi^	60.986 (12)	Mo3^iii^—Sc—Sc^iii^	163.78 (9)
Mo3^v^—Mo3—Mo2^vi^	89.795 (12)	Se3^iii^—Sc—Sc^iii^	110.81 (7)
Mo3^vi^—Mo3—Mo2^vi^	61.256 (13)	Se4^viii^—Sc—Sc^ii^	49.97 (2)
Mo2^ix^—Mo3—Mo2^vi^	112.82 (2)	Se4—Sc—Sc^ii^	49.97 (2)
Se2^viii^—Mo3—Mo2	145.46 (2)	Se3^ii^—Sc—Sc^ii^	117.39 (6)
Se2—Mo3—Mo2	57.907 (12)	Se2^iii^—Sc—Sc^ii^	114.00 (7)
Se3^v^—Mo3—Mo2	60.350 (12)	Se2^xviii^—Sc—Sc^ii^	114.00 (7)
Se3—Mo3—Mo2	118.538 (13)	Mo3^iii^—Sc—Sc^ii^	103.78 (9)
Mo3^v^—Mo3—Mo2	59.495 (13)	Se3^iii^—Sc—Sc^ii^	50.81 (7)
Mo3^vi^—Mo3—Mo2	88.794 (12)	Sc^iii^—Sc—Sc^ii^	60.0

Symmetry codes: (i) *x*−*y*, *x*, −*z*+1; (ii) −*y*, *x*−*y*, *z*; (iii) −*x*+*y*, −*x*, *z*; (iv) *y*, −*x*+*y*, −*z*+1; (v) −*y*+1, *x*−*y*, *z*; (vi) −*x*+*y*+1, −*x*+1, *z*; (vii) −*x*+*y*+1, −*x*, *z*; (viii) *x*, *y*, −*z*+3/2; (ix) −*x*+*y*+1, −*x*+1, −*z*+3/2; (x) −*y*, *x*−*y*−1, *z*; (xi) −*x*+1, −*y*, −*z*+1; (xii) *y*+1, −*x*+*y*+1, −*z*+1; (xiii) *x*−*y*+1, *x*, −*z*+1; (xiv) −*x*+1, −*y*+1, −*z*+1; (xv) *y*+1, −*x*+*y*, −*z*+1; (xvi) −*x*, −*y*, −*z*+1; (xvii) *x*−*y*+1, *x*+1, −*z*+1; (xviii) −*x*+*y*, −*x*, −*z*+3/2; (xix) −*y*, *x*−*y*−1, −*z*+3/2.
